# The Efficacy and Safety of the Chinese Herbal Formula, JTTZ, for the Treatment of Type 2 Diabetes with Obesity and Hyperlipidemia: A Multicenter Randomized, Positive-Controlled, Open-Label Clinical Trial

**DOI:** 10.1155/2018/9519231

**Published:** 2018-04-01

**Authors:** Xiaotong Yu, Lipeng Xu, Qiang Zhou, Shengping Wu, Jiaxing Tian, Chunli Piao, Hailong Guo, Jun Zhang, Liping Li, Shentao Wu, Meizhen Guo, Yuzhi Hong, Weirong Pu, Xiyan Zhao, Yang Liu, Bing Pang, Zhiping Peng, Song Wang, Fengmei Lian, Xiaolin Tong

**Affiliations:** ^1^Graduate School, Beijing University of Chinese Medicine, Beijing 100029, China; ^2^Guang'anmen Hospital, China Academy of Chinese Medical Sciences, Beijing 100053, China; ^3^Affiliated Hospital of Changchun University of TCM, Changchun, Jilin 130021, China; ^4^Tianjin Dagang Hospital, Tianjin 300270, China; ^5^Yichang Yiling Hospital, Hubei 443100, China; ^6^Baoding Hospital of TCM, Baoding, Hebei 071000, China; ^7^First Teaching Hospital of Tianjin University of Traditional Chinese Medicine, Tianjin 300193, China; ^8^She County Hospital, Handan, Hebei 056400, China; ^9^Hangzhou Hospital of TCM, Hangzhou, Zhejiang 310007, China; ^10^Qinghai Hospital of TCM, Xining, Qinghai 810000, China

## Abstract

**Background and Aim:**

Studies have shown an increasing number of type 2 diabetes (T2D) patients with concomitant obesity and hyperlipidemia syndromes, resulting from relevant metabolic disorders. However, there are few medications and therapies, which can thoroughly address these issues. Therefore, the current study evaluated the efficacy and safety of using JTTZ, a Chinese herbal formula, to treat T2D with obesity and hyperlipidemia.

**Methods:**

A total of 450 participants with T2D (HbA1c ≥ 7.0%; waist circumference ≥ 90 cm and 80 cm in males and females, resp.; and triglycerides (TG) ≥ 1.7 mmol/L) were randomly assigned, in equal proportions, to two groups in this multicenter randomized, positive-controlled, open-label trial. One group received JTTZ formula, and the other received metformin (MET) for 12 consecutive weeks. The primary efficacy outcomes were changes in HbA1c, TG, weight, and waist circumference. Adverse reactions and hypoglycemia were monitored.

**Results:**

HbA1c decreased by 0.75 ± 1.32% and 0.71 ± 1.2% in the JTTZ and MET groups, respectively, after 12 weeks of treatment. TG levels in the JTTZ and MET groups were reduced by 0.64 ± 2.37 mmol/L and 0.37 ± 2.18 mmol/L, respectively. Weight was decreased by 2.47 ± 2.71 kg in the JTTZ group and by 2.03 ± 2.36 kg in the MET group. JTTZ also appeared to alleviate insulin resistance and increase HOMA-*β*. In addition, symptoms were significantly relieved in participants in the JTTZ group compared to those in the MET group. One case of hypoglycemia was reported in the MET group. No severe adverse events were reported in either group.

**Conclusions:**

The JTTZ formula led to safe and significant improvements in the blood glucose, blood lipids, and weight levels; relieved symptoms; and enhanced *β* cell function for T2D patients with obesity and hyperlipidemia. The JTTZ formula has shown that it could potentially be developed as an alternative medicine for patients with T2D, particularly those who cannot tolerate metformin or other hypoglycemic drugs. This trial was registered with Clinicaltrials.gov NCT01471275.

## 1. Introduction

Type 2 diabetes (T2D) is a complex progressive metabolic disorder, which poses a significant threat to human health and accounts for more than 91% of all diabetes cases. In 2015, it was estimated that 415 million adults suffered from diabetes mellitus, representing 9.1% of the global adult population. China has the largest number of diabetes patients worldwide—up to 109 million [1]. In addition, with the modernization of lifestyles and changes in dietary habits, 60–70% of those with diabetes also have obesity and 70–80% have hyperlipidemia [2, 3]. T2D patients with concomitant obesity and hyperlipidemia have more difficulty in controlling blood sugar levels, a higher risk of cardiovascular events, and a higher incidence of mortality [4].

Treatments for T2D are aimed at balancing the antihyperglycemic efficacy, risk of inducing hypoglycemia, risk of weight gain, tolerability, and other adverse effects. When diabetes is associated with obesity and hyperlipidemia, it is recommended that the algorithm for the comprehensive management of T2D be used [5]. However, studies have shown that, in addition to having side effects, synthetic drugs cannot effectively control glucose levels in some patients [6–8]. Furthermore, for those with multiple metabolic disorders, comprehensive and effective treatments are sorely lacking [9].

In previous studies, Chinese herbal medicines have been shown to effectively reduce blood glucose [10–12]. However, few studies have been conducted examining the used-alone effects of the traditional Chinese formula on those with T2D and concomitant abdominal obesity and hyperlipidemia. JTTZ formula is derived from a classical formula—Dahuang Huanglian Xiexin Decoction, which was recorded in the *Treatise on Febrile Diseases* (Han Dynasty, 200 AD) and used for stomach heat syndrome in ancient China. We have used JTTZ formula in our clinical application for thousands of patients, which was satisfying in lowering blood glucose, blood lipids, and weight levels. In the current study, the preliminary efficacy and safety of the JTTZ formula were evaluated for the treatment of T2D with obesity and hyperlipidemia and were also compared to those of the first-line antidiabetic oral agent, metformin.

## 2. Materials and Methods

### 2.1. Study Design

The research was a multicenter randomized, positive-controlled, open-label clinical trial, which included a 4-week screening period and a 12-week treatment period. The study was approved by the Ethics Committee of Guang'anmen Hospital of the China Academy of Chinese Medical Sciences (number eighty-sixth 2011) and was conducted in accordance with the principles of the Declaration of Helsinki. Participants were recruited by nine branch centers, and all participants signed informed consents before the study. The registration number is NCT01471275.

### 2.2. Subject Enrollment

Patients were diagnosed with T2D via fasting plasma glucose (FPG) tests and 75 g oral glucose tolerance tests (OGTT). Hyperlipidemia and obesity were diagnosed via measurements of triglyceride level (TG) and waist circumference (WC).

#### 2.2.1. Inclusion Criteria

(1) Informed consent was signed. (2) WC was ≥90 cm in males and ≥80 cm in females. (3) Patients were newly diagnosed with T2D, according to the 1999 World Health Organization criteria (OGTT test, FPG ≥ 7 mmol/L, or 2 h postprandial plasma glucose (2 h PG) ≥ 11.1 mmol/L), after a screening period (4-week diet and exercise therapy), and had not previously received pharmacological treatment. (4) Patients had a glycosylated hemoglobin (HbA1c) ≥ 7.0% and an FPG < 13.9 mmol/L. (5) TG levels were ≥1.7 mmol/L but <5.65 mmol/L. (6) Patients were between 18 and 70 years of age.

#### 2.2.2. Exclusion Criteria

The exclusion criteria are as follows: (1) patients who had used insulin therapy or previously undergone continuous treatment for diabetes lasting 3 months or longer, including Chinese and western medicine, physical therapy, psychological therapy, and health food; (2) patients who received hypoglycemic or hypolipidemic medication within the previous month; (3) patients with diabetic complications (serious heart, lung, liver, kidney, or brain complications) or patients with diabetes accompanied by other serious primary diseases; (4) a systolic blood pressure (SBP) ≥ 160 mmHg or diastolic blood pressure (DBP) ≥ 100 mmHg; (5) patients that had diabetic ketosis, diabetic ketoacidosis, or severe infections within the previous month; (6) patients with psychiatric disease; (7) patients who were pregnant, who were planning to become pregnant, or who were breast-feeding; (8) patients who were allergic to the JTTZ formula or any of its major components or who had allergic constitutions; (9) patients involved in other clinical trials currently or within the previous month; (10) alcohol abuse and/or use of psychoactive substances or drug abuse and dependency within the past 5 years; (11) patients who were less likely to complete the study, according to the researcher's judgment, such as those being lost to follow-up due to frequent changes in work or living environments; (12) fluctuating dosage and category of antihypertensive drugs; (13) patients taking diet pills or health food that had an effect on body weight; (14) patients with impaired liver and renal function (alanine aminotransferase (ALT) and aspartate aminotransferase (AST) 2 times greater than the upper limit of normal and serum creatinine (Cr) greater than the upper limit of normal); and (15) patients that had asymptomatic hypoglycemia.

#### 2.2.3. Power Calculation

The dose selection of metformin follows the “small starting dose” principle [13] and the direction for use of metformin hydrochloride enteric-coated tablets. Based on the literature, after removal of the placebo effect, the HbA1c level generally reduced to 0.7% by treatment using 750 mg/d metformin for 12 weeks of intervention [14, 15]. Thus, we need to enroll 180 subjects per group to ensure a 5% alpha level and an 80% power. By considering factors such as dropouts, 225 subjects were recruited per group.

#### 2.2.4. Withdrawal Criteria

(1) Participants experienced specific physiological changes or serious diabetic complications (such as diabetic ketoacidosis, hyperosmolar nonketotic syndrome, lactic acidosis, and hypoglycemic coma). (2) Participants had poor compliance, with test medication use less than 80% or more than 120% of the prescribed dose. (3) Participants had violated the protocol (e.g., taking another oral hypoglycemic agent). (4) Participants withdrew from the study voluntarily. Participants had the right to withdraw from the trial voluntarily, according to the informed consent. Reasons for withdrawal were noted as much as possible, and the efficacy and any adverse reactions were recorded.

### 2.3. Drug Administration

The JTTZ formula was composed of 8 herbs, including Luhui (*Aloe vera*), Huanglian (*Coptis chinensis*), Zhimu (Rhizoma Anemarrhenae), Hongqu (red yeast rice), Kugua (*Momordica charantia*), Danshen (*Salvia miltiorrhiza*), Wuweizi (*Schisandra chinensis*), and Ganjiang (dried ginger). The dose was given in the form of boil-free granules. Herbs were boiled, filtered, concentrated into a cream, and dried into granules. The Jiangyin Tianjiang Pharmaceutical Co. Ltd. (Jiangsu, China) provided all the herbs, and herbs were quality controlled in accordance with the *Pharmacopoeia of the People's Republic of China* (2010). The manufacturer was not involved in the design or analysis of this study. The granules were packed into individual bags. Participants in the JTTZ group took the granules orally 2 times per day, at mealtimes. The method of taking the medication is just like drinking an instant coffee: put the granules into a cup, and then pour some hot water and stir well.

Participants in the control group received metformin hydrochloride enteric-coated tablets, which were all provided and quality controlled by Beijing Liling Hengtai Pharmaceutical Co. Ltd. (Approval number: H11021560; Batch number: 110508). Participants in the control group took 0.25 g metformin tablets orally 3 times per day. Participants in both groups underwent treatment for 12 weeks.

### 2.4. Chemical Analysis of the JTTZ Formula

**Figure 1:**
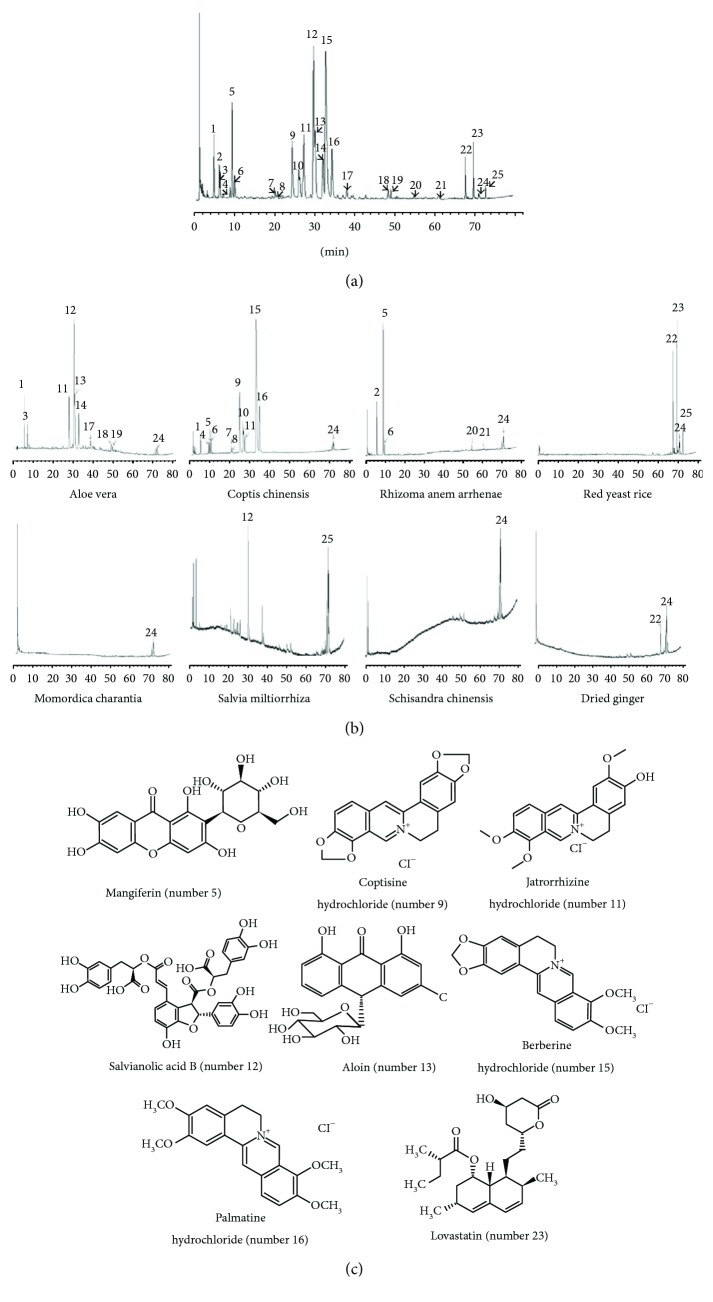
High-performance liquid chromatography (HPLC) analysis of the JTTZ formula. (a) Representative HPLC chromatogram of the JTTZ formula. (b) Representative HPLC chromatograms of single granules in the JTTZ formula. (c) Chemical structures of the identified compounds in the JTTZ formula, corresponding to the peak numbers indicated in the chromatograms.

The chemical composition of the JTTZ formula was analyzed using high-performance liquid chromatography (HPLC). The separation was carried out on a Merck Chromolith RP-18e column (4.6 × 100 mm inner diameter). For HPLC analysis, a 5 *μ*L test sample was injected into the column and eluted at a constant flow rate of 2 mL/min. Water with 0.02% phosphoric acid (mobile phase A) and acetonitrile (mobile phase B) was used. Composition was determined at a wavelength of 237 nm. The JTTZ formula powder was dissolved in methanol. All solutions were filtered through 0.45 *μ*m nylon membrane Millex syringe filters before use. Chemical structures of the eight major compounds (mangiferin, coptisine hydrochloride, jatrorrhizine hydrochloride, salvianolic acid B, aloin, berberine hydrochloride, palmatine hydrochloride, and lovastatin) were identified in the finished dose. Representative chromatograms of the JTTZ formula and the single granule and chemical structures of the major compounds are shown in Figure 1.

### 2.5. Evaluation of Efficacy

The primary efficacy outcomes were changes in HbA1c, TG, weights, and WC. Secondary efficacy outcomes were the target achievement rates for HbA1c and TG; changes in FPG, 2 h PG, insulin resistance index (HOMA-IR), *β* cell function index (HOMA-*β*), total cholesterol (TC), low-density lipoprotein cholesterol (LDL-C), body mass index (BMI), and hip circumferences (HC); and symptom scores of the participants. Measurements of FPG, weight, WC, and HC were done every 4 weeks; other assessments were performed before and after the 12-week treatment phase.

Evaluation of 2 h PG was done using a standard 75 g OGTT. Body mass index (kg/m^2^) was calculated according to height and weight. Plasma insulin level was measured, and the homeostatic model assessment was performed to quantify HOMA-IR and HOMA-*β*.

The remaining whole blood was collected at the branch center, after monitoring of HbA1c. The remaining serum was collected and tested again at the central laboratory, after blood lipid levels were tested at the branch center. Serum insulin was collected and tested at the central laboratory. Specimens unable to be transported to the central laboratory within 24 hours were frozen at −20°C and cold chain transport of unification.

### 2.6. Participant Symptom Scores

Symptoms of gastrointestinal excess heat syndrome are thoracoabdominal distension, abdominal fullness, constipation, dry and bitter mouth, foul breath, thirst, drinking cold water, increased eating with rapid hunger, red tongue with yellow coating, and powerful and rapid pulse. The main symptoms reported were abdominal distension and fullness, as well as constipation. Each symptom was divided into 4 levels and scored as 0, 2, 4, and 6 (with 6 being the most severe). Secondary symptoms were other symptoms of gastrointestinal excess heat syndrome. Tongue and pulse presentations were divided into 2 categories (0 being no and 2 being yes), and the rest of the symptoms were divided into 4 levels and were quantified as scores 0, 1, 2, and 3 (3 being the most severe).

### 2.7. Evaluation of Safety

Vital signs; routine blood, urine, and stool samples; electrocardiograms; and liver and renal function (ALT, AST, Cr, and BUN) were all used to evaluate safety. Safety indexes were tested every 4 weeks. Hypoglycemia is divided into severe hypoglycemia and nonsevere hypoglycemia. Severe hypoglycemia refers to a disturbance of consciousness that requires help from others. Neurological symptoms are significantly improved or disappeared after hypoglycemia has been corrected. Nonsevere hypoglycemia refers to diabetic patients with blood glucose levels ≤ 3.9 mmol/L after taking medication, which may have hypoglycemia symptoms, but no disturbance of consciousness [16]. Adverse events and the occurrence of hypoglycemia were recorded and managed, and correlations with the medications were analyzed. The safety procedures were in place when the local Medical Ethics Commission in China approved the proposal. The Data and Safety Monitoring Board was responsible for the oversight of all issues related to the safety of the study subjects.

### 2.8. Random Design

An Interactive Web Response System designed by the Clinical Evaluation Center of China Academy of Chinese Medical Sciences was used in this study to produce a random code for the trial group and control group in equal proportions. Each participant was assigned a random number, and the drugs were distributed according to the number. Case report forms were obtained at the end of the experiment, and the data were analyzed by an independent third-party statistical agency.

### 2.9. Statistical Analysis

Data was entered using the EpiData3.1 software (EpiData Association, Odense, Denmark). Data was analyzed using the SPSS 20.0 software (SPSS Inc., Chicago, IL, USA). Measurement data is described as mean ± standard deviation. A *t*-test or Wilcoxon rank sum test was used to analyze differences between two groups. In each group, changes between baseline (before treatment) and posttreatment (end of the study) were analyzed using a comparative *t*-test; the primary endpoints were compared using a covariance analysis model with the baseline as the covariate. Enumeration data was described using frequency. Changes, which occurred during the period before and after the treatment, were compared using the CMH *χ*^2^ test; the incidences of adverse events between the two groups were compared by the CMH *χ*^2^ test or Fisher exact probability test. All statistical tests were two-sided. A significance level of 5% was used, and 95% confidence intervals were calculated.

## 3. Results

**Figure 2:**
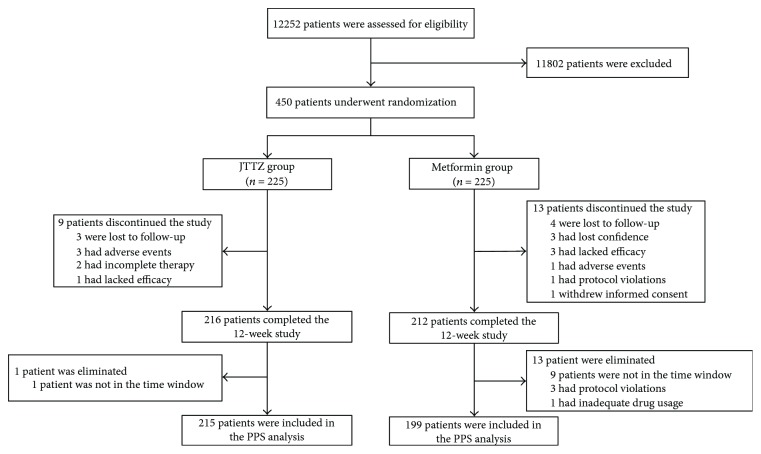
Flow diagram of participant screening, randomization, and treatment.

A total of 12252 subjects underwent initial screening. According to the inclusion and exclusion criteria, after a 4-week screening period (diet and exercise therapy), 450 subjects were enrolled and randomly divided into the trial group (JTTZ; *n* = 225) and the control group (metformin (MET); *n* = 225). In the JTTZ group, 216 subjects completed the study and 215 were included in the PPS analysis. In the MET group, 212 subjects completed the study and 199 were included in the PPS analysis (Figure 2).

### 3.1. Baseline

The baseline characteristics of the subjects are shown in Table 1. The values showed no differences between groups.

### 3.2. Effectiveness

#### 3.2.1. Effects on the Change of HbA1c and TG Levels

At week 12, the HbA1c levels (%) in the JTTZ and MET groups were 7.51 ± 1.44 and 7.57 ± 1.42, respectively. HbA1c level was significantly lower in both groups, compared to that at baseline (*P* < 0.001), with a 0.75 ± 1.32 reduction (95% CI = 0.58–0.93) in the JTTZ group and a 0.71 ± 1.2 reduction (95% CI = 0.54–0.87) in the MET group.

**Figure 3:**
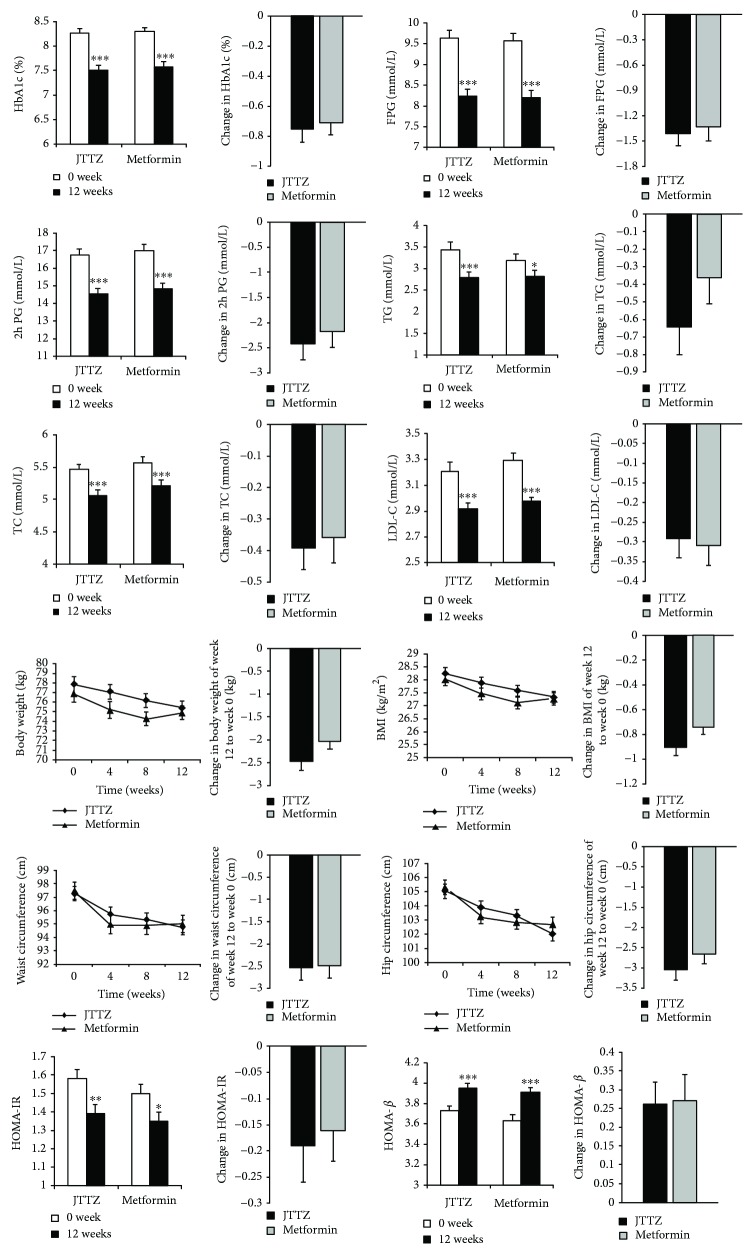
Changes in the T2D levels, hyperlipidemia levels, obesity levels, and pancreatic islet function levels of participants receiving JTTZ formula and metformin. Data presented as mean ± SE. ^∗^*P* < 0.05, ^∗∗^*P* < 0.01, and ^∗∗∗^*P* < 0.001.

At week 12, the TG levels (mmol/L) in the JTTZ group and MET groups were 2.79 ± 1.86 and 2.82 ± 1.91, respectively. The level was significantly reduced by 0.64 ± 2.37 (*P* < 0.001) in the JTTZ group and by 0.37 ± 2.18 (*P* < 0.05) in the MET group, compared to that at baseline. The 95% CI was 0.32–0.96 in the JTTZ group and 0.06–0.67 in the MET group (Table 2, Figure 3).

#### 3.2.2. Effects on HbA1c Target Achievement Rate and the Levels of Glucose

The HbA1c value in 45.1% (97/215) of the subjects in the JTTZ group and 42.7% (85/199) in the MET group achieved the target, using HbA1c ≤ 7.0% as the target achievement. There was no statistical difference between the two groups (CMH *χ*^2^ = 0.039; *P* = 0.843).

The FPG levels (mmol/L) in the JTTZ and MET groups were 8.24 ± 2.35 and 8.21 ± 2.23, respectively, at week 12. The level was significantly reduced by 1.4 ± 2.4 in the JTTZ group and by 1.33 ± 2.33 in the MET group compared to that at baseline (*P* < 0.001).

The 2 h PG levels (mmol/L) in the JTTZ and MET groups were 14.52 ± 4.73 and 14.83 ± 4.32, respectively, at week 12. The level was significantly reduced by 2.42 ± 4.53 in the JTTZ group and by 2.18 ± 4.48 in the MET group (*P* < 0.001) (Table 2, Figure 3).

#### 3.2.3. Effects on TG Target Achievement Rate and the Levels of TC and LDL-C

The TG value in 27.4% (59/215) of the subjects in the JTTZ group and 27.1% (54/199) of the subjects in the MET group achieved the target, when using TG ≤ 1.7 mmol/L as the target achievement. There was no statistical difference between the two groups (CMH *χ*^2^ = 0.035, *P* = 0.852).

The TC levels (mmol/L) in the JTTZ and MET groups were 5.06 ± 1.00 and 5.21 ± 1.00, respectively, at week 12. The level was significantly reduced by 0.39 ± 0.99 in the JTTZ group and by 0.36 ± 1.16 in the MET group (*P* < 0.001), compared to that at baseline.

The LDL-C levels (mmol/L) in the JTTZ and MET groups were 2.92 ± 0.73 and 2.98 ± 0.76, respectively, at week 12. Compared with the baseline, both groups had a very significant difference, respectively (*P* < 0.001). The reduced levels of LDL-C showed no obvious differences between the two groups (Table 2, Figure 3).

#### 3.2.4. Effects on Weight, BMI, Waist Circumference, and Hip Circumference

Table 2 and Figure 3 show the levels and changes in weight, BMI, waist circumference, and hip circumference at weeks 0, 4, 8, and 12. Compared with the baseline, both groups had a very significant difference at week 12 (*P* < 0.001). The JTTZ group decreased more than did the MET group in each project; however, there was no obvious difference between the changes (Table 2, Figure 3).

#### 3.2.5. Effects on HOMA-IR and HOMA-*β*

At week 12, the HOMA-IR levels in the JTTZ and MET groups were 1.39 ± 0.68 and 1.35 ± 0.67, respectively. The level was significantly reduced by 0.19 ± 0.91 in the JTTZ group (*P* < 0.01) and by 0.16 ± 0.82 (*P* < 0.05) in the MET group, compared to that at baseline.

The HOMA-*β* levels in the JTTZ and MET groups were 3.95 ± 0.76 and 3.91 ± 0.73, respectively, at week 12. Compared with the baseline, both groups had a very significant difference, respectively (*P* < 0.001). The increased levels of HOMA-*β* showed no obvious differences between the two groups (Table 2, Figure 3).

#### 3.2.6. Effects on Participant Symptoms

Participant symptom score level in the JTTZ and MET groups were 5.80 ± 4.05 and 8.57 ± 4.40, respectively, at week 12. The level was reduced by 5.23 ± 4.20 in the JTTZ group and by 2.21 ± 4.11 in the MET group, compared to that at baseline (*P* < 0.001). The 95% CI was 4.67–5.80 in the JTTZ group and 1.64–2.79 in the MET group. The level decreased significantly more in the JTTZ group than in the MET group (*P* < 0.001).

**Figure 4:**
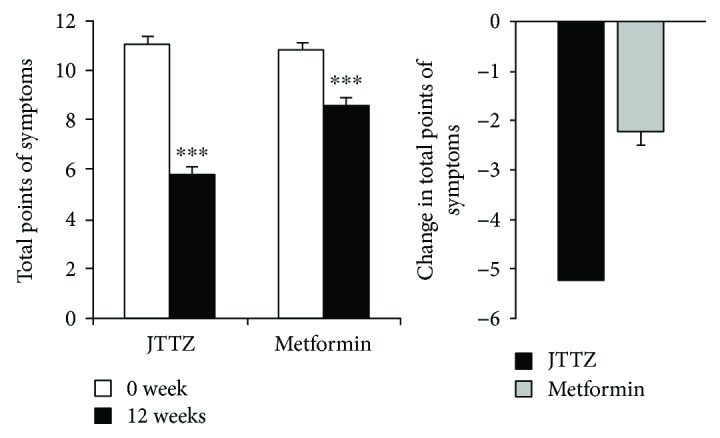
Changes in the total symptom score. Data are presented as mean ± SE. ^∗∗∗^*P* < 0.001.

After the 12-week treatment, the following symptoms had improved more in the JTTZ group than in the MET group: thoracoabdominal distension, constipation, bitter mouth, halitosis, increased eating with rapid hunger, red tongue and yellow tongue coating, and powerful and rapid pulse (*P* < 0.05). Comparison of symptom scores and symptom disappearance rates is shown in Table 3 and Figure 4.

### 3.3. Safety Evaluation

No severe adverse events were reported during the trial. Thirteen nonsevere adverse events (6.0%) were reported in the JTTZ group, and nine nonsevere adverse events (4.5%) were reported in the MET group (*P* = 0.490). There were two cases of transient slight ALT and AST elevation in the both groups. One case of nonsevere hypoglycemia was reported in the MET group. No abnormal ECGs, renal function tests (serum creatinine), or routine blood tests were observed during the trial.

## 4. Discussion

Metformin is a very effective antihyperglycemic and is recommended as a first-line choice for the treatment of T2D [5]. In addition, metformin promotes weight loss, as well as a modest decrease in blood lipid levels [17–19], and has robust cardiovascular safety [20, 21]. However, some patients cannot tolerate the gastrointestinal-related side effects of metformin [22]. The incidence of gastrointestinal adverse events associated with 500 mg/d and 1000 mg/d metformin is about 15% and 28%, respectively [14, 23]. It has also been suggested that metformin be stopped when estimated glomerular filtration rate (eGFR) is less than 30 mL/min/1.73 m^2^ due to the risk of lactic acidosis [24, 25]. Furthermore, metformin may lead to a vitamin B12 deficiency [26, 27].

In this current study, after 12-week treatment, significant reductions in blood glucose, blood lipid, and weight levels were observed in both the JTTZ formula group and the metformin group. The HbA1c level was decreased by 0.75% in the JTTZ group, which had no obvious difference when compared to the MET group with a 0.71% reduction. Compared to baseline, both groups had significant improvements in HOMA-*β* levels and decreases in HOMA-IR levels. The TG levels were significantly reduced by 0.64 mmol/L in the JTTZ group and by 0.37 mmol/L in the MET group. Weight levels in the JTTZ and MET groups were decreased by 2.47 kg and 2.03 kg, respectively. Although there was no significant difference between the changes, using the JTTZ formula provided a greater reduction than using metformin in progression. Furthermore, in contrast to metformin, the JTTZ formula significantly improved patient symptoms. One case of hypoglycemia was reported in the MET group, and both groups had no severe adverse events.

Previous studies demonstrated that many active ingredients in the traditional Chinese medicine formula, JTTZ, can also effectively manage diabetes, obesity, and dyslipidemia. Some researchers have shown that mangiferin in Rhizoma Anemarrhenae has a positive effect on weight loss, lowering blood sugar and fat, decreasing arteriosclerosis, and improving cardiovascular complications [28–30]. Berberine in Rhizoma has been shown to reduce HbA1c by 1%, TG by 0.9 mmol/L, and TC by 0.96 mmol/L after 3 months of treatment [31]. *Aloe vera* gel extract resulted in a reduction in fasting blood glucose, hepatic transaminases, plasma and tissue cholesterol, triglycerides, free fatty acids, and phospholipids in diabetic rats after 21 days of treatment [32]. Moreover, *Salvia miltiorrhiza* has cardiovascular protective effects, and *Schisandra chinensis* has antioxidant and hepatoprotective effects [33, 34].

A comprehensive management of glycemic control and cardiovascular risk factors such as lipids and weight is crucial [35, 36]. This study indicates that the JTTZ formula safely and effectively reduced blood sugar levels, lowered blood lipid levels, promoted weight loss, enhanced *β* cell function, and relieved symptoms. It may be used as an alternative medicine for those with T2D and concomitant obesity and hyperlipidemia, which is supposed to be paid more attention in the treatments of type 2 diabetes, particularly in patients who cannot tolerate metformin or other hypoglycemic drugs. The limitation of this study was that the 12-week duration of the trial was relatively short. Maybe longer-term observation and mechanism research will demonstrate the formula effects more clearly.

## Figures and Tables

**Table 1:** Baseline characteristics of participants receiving JTTZ formula and metformin.

Characteristics	JTTZ group (*n* = 215)	MET group (*n* = 199)	*P* values
*General information*			
Age (years)	52.82 ± 9.01	52.90 ± 8.52	0.922
Sex (male/female)	104/111	98/101	0.859
Past medical history (no/yes)	77/138	83/116	0.218
Current medications (no/yes)	125/90	128/71	0.197
*Physical examinations*			
Systolic BP (mmHg)	129.55 ± 12.36	131.11 ± 11.95	0.196
Diastolic BP (mmHg)	82.80 ± 8.03	82.62 ± 8.24	0.823
Heart rate (beat/min)	74.80 ± 8.47	73.31 ± 7.84	0.064
Height (cm)	165.81 ± 8.24	165.41 ± 8.80	0.638
Weight (kg)	77.82 ± 12.08	76.86 ± 12.06	0.421
BMI (kg/m^2^)	28.24 ± 3.31	28.01 ± 3.22	0.485
Waist circumference (cm)	97.25 ± 8.09	97.47 ± 9.24	0.800
Hip circumference (cm)	105.05 ± 7.14	105.31 ± 7.36	0.717
*Efficacy indicators*			
HbA1c (%)	8.26 ± 1.35	8.28 ± 1.33	0.908
FPG (mmol/L)	9.64 ± 2.59	9.56 ± 2.39	0.744
2 h PG (mmol/L)	16.76 ± 4.93	17.00 ± 4.72	0.610
Fasting insulin (pmol/L)	103.89 ± 97.73	100.88 ± 131.62	0.796
TC (mmol/L)	5.46 ± 1.24	5.56 ± 1.36	0.412
TG (mmol/L)	3.43 ± 2.83	3.18 ± 2.14	0.321
LDL-C (mmol/L)	3.21 ± 0.96	3.29 ± 0.90	0.429
HOMA-IR	1.58 ± 0.72	1.5 ± 0.75	0.286
HOMA-*β*	3.73 ± 0.76	3.63 ± 0.78	0.176
*Cardinal symptoms*			
Total symptom points	11.03 ± 4.93	10.78 ± 4.63	0.598

Data presented as mean ± standard deviation. Current medications: participants were taking or not taking other medications during the study period; BMI: body mass index; HbA1c: glycosylated hemoglobin; FPG: fasting plasma glucose; 2 h PG: 2 h postprandial plasma glucose; TC: total cholesterol; TG: triglycerides; LDL-C: low-density lipoprotein cholesterol; HOMA-IR: insulin resistance index; HOMA-*β*: *β* cell function index.

**Table 2:** Changes in the T2D levels, hyperlipidemia levels, obesity levels, and pancreatic islet function levels of participants receiving JTTZ formula and metformin.

	JTTZ group				MET group				*P* values: week 12 in the JTTZ group versus MET group	*P* values: change in the JTTZ group versus MET group
	Baseline	Week 12	Change from baseline to week 12	*P* values: baseline versus week 12	Baseline	Week 12	Change from baseline to week 12	*P* values: baseline versus week 12		
*T2D levels*										
HbA1c (%)	8.26 ± 1.35	7.51 ± 1.44	−0.75 ± 1.32	0.000	8.28 ± 1.33	7.57 ± 1.42	−0.71 ± 1.2	0.000	0.639	0.700
FPG (mmol/L)	9.64 ± 2.59	8.24 ± 2.35	−1.4 ± 2.4	0.000	9.56 ± 2.39	8.21 ± 2.23	−1.33 ± 2.33	0.000	0.865	0.781
2 h PG (mmol/L)	16.76 ± 4.93	14.52 ± 4.73	−2.42 ± 4.53	0.000	17.00 ± 4.72	14.83 ± 4.32	−2.18 ± 4.48	0.000	0.500	0.606
*Hyperlipidemia levels*										
TG (mmol/L)	3.43 ± 2.83	2.79 ± 1.86	−0.64 ± 2.37	0.000	3.18 ± 2.14	2.82 ± 1.91	−0.37 ± 2.18	0.018	0.889	0.227
TC (mmol/L)	5.46 ± 1.24	5.06 ± 1.00	−0.39 ± 0.99	0.000	5.56 ± 1.36	5.21 ± 1.00	−0.36 ± 1.16	0.000	0.147	0.764
LDL-C (mmol/L)	3.21 ± 0.96	2.92 ± 0.73	−0.29 ± 0.79	0.000	3.29 ± 0.90	2.98 ± 0.76	−0.31 ± 0.71	0.000	0.401	0.812
*Obesity levels*										
Weight (kg)	77.82 ± 12.08	75.34 ± 12.05	−2.47 ± 2.71	0.000	76.86 ± 12.06	74.83 ± 12.00	−2.03 ± 2.36	0.000	0.666	0.093
BMI (kg/m^2^)	28.24 ± 3.31	27.33 ± 3.34	−0.9 ± 0.99	0.000	28.01 ± 3.22	27.27 ± 3.21	−0.74 ± 0.86	0.000	0.844	0.096
Waist circumference (cm)	97.25 ± 8.09	94.73 ± 8.29	−2.52 ± 4.29	0.000	97.47 ± 9.24	94.98 ± 9.03	−2.49 ± 3.86	0.000	0.774	0.946
Hip circumference (cm)	105.05 ± 7.14	102.02 ± 7.23	−3.04 ± 3.86	0.000	105.31 ± 7.36	102.67 ± 7.16	−2.65 ± 3.51	0.000	0.359	0.225
*Pancreatic islet function levels*										
HOMA-IR	1.58 ± 0.72	1.39 ± 0.68	−0.19 ± 0.91	0.005	1.5 ± 0.75	1.35 ± 0.67	−0.16 ± 0.82	0.010	0.626	0.930
HOMA-*β*	3.73 ± 0.76	3.95 ± 0.76	0.26 ± 0.82	0.000	3.63 ± 0.78	3.91 ± 0.73	0.27 ± 0.87	0.000	0.638	0.752

Data presented as mean ± standard deviation. HbA1c: glycosylated hemoglobin; FPG: fasting plasma glucose; 2 h PG: 2 h postprandial plasma glucose; TC: total cholesterol; TG: triglycerides; LDL-C: low-density lipoprotein cholesterol; BMI: body mass index; HOMA-IR: insulin resistance index; HOMA-*β*: *β* cell function index.

**Table 3:** The changes in patients' symptoms.

	JTTZ group	Metformin group	*P* values
*Total symptom score*			
Week 0	11.03 ± 4.93	10.78 ± 4.63	0.598
Week 12	5.80 ± 4.05	8.57 ± 4.40	0.000
Week 12–week 0	−5.23 ± 4.20	−2.21 ± 4.11	0.000
Paired sample *t*-test (*P*)	0.000	0.000	
*Symptom disappearance rate*			
Thoracoabdominal distension	55 (44.4%)	35 (27.8%)	0.006
Abdominal fullness	74 (54.8%)	52 (46.0%)	0.168
Constipation	62 (72.9%)	26 (31.7%)	0.000
Dry mouth	55 (33.7%)	41 (25.9%)	0.128
Bitter mouth	49 (41.2%)	33 (28.4%)	0.041
Halitosis	46 (45.1%)	18 (21.7%)	0.001
Thirst and drinking cold water	35 (29.4%)	28 (25.0%)	0.453
Increased eating with rapid hunger	57 (46.7%)	33 (30.8%)	0.014
Red tongue and yellow tongue coating	51 (29.8%)	28 (18.8%)	0.023
Power and rapid pulse	71 (50.0%)	34 (27.6%)	0.000

## References

[1] Ogurtsova K., da Rocha Fernandes J. D., Huang Y. (2017). IDF diabetes atlas: global estimates for the prevalence of diabetes for 2015 and 2040. *Diabetes Research and Clinical Practice*.

[2] Selvin E., Parrinello C. M., Sacks D. B., Coresh J. (2014). Trends in prevalence and control of diabetes in the United States, 1988–1994 and 1999–2010. *Annals of Internal Medicine*.

[3] Preis S. R., Pencina M. J., Hwang S. J. (2009). Trends in cardiovascular disease risk factors in individuals with and without diabetes mellitus in the Framingham Heart Study. *Circulation*.

[4] Benjamin E. J., Blaha M. J., Chiuve S. E. (2017). Heart disease and stroke statistics–2017 update: a report from the American Heart Association. *Circulation*.

[5] Garber A. J., Abrahamson M. J., Barzilay J. I. (2017). Consensus statement by the American Association of Clinical Endocrinologists and American College of Endocrinology on the comprehensive type 2 diabetes management algorithm – 2017 executive summary. *Endocrine Practice*.

[6] American Diabetes Association (2016). Standards of medical care in diabetes. *Diabetes Care*.

[7] Ji L. N., Lu J. M., Guo X. H. (2013). Glycemic control among patients in China with type 2 diabetes mellitus receiving oral drugs or injectables. *BMC Public Health*.

[8] Dodd A. H., Colby M. S., Boye K. S., Fahlman C., Kim S., Briefel R. R. (2009). Treatment approach and HbA_1c_ control among US adults with type 2 diabetes: NHANES 1999–2004. *Current Medical Research and Opinion*.

[9] Alberti K. G., Zimmet P., Shaw J. (2006). Metabolic syndrome–a new world-wide definition. A consensus statement from the International Diabetes Federation. *Diabetic Medicine*.

[10] Lian F., Tian J., Chen X. (2015). The efficacy and safety of Chinese herbal medicine Jinlida as add-on medication in type 2 diabetes patients ineffectively managed by metformin monotherapy: a double-blind, randomized, placebo-controlled, multicenter trial. *PLoS One*.

[11] Xu J., Lian F., Zhao L. (2015). Structural modulation of gut microbiota during alleviation of type 2 diabetes with a Chinese herbal formula. *ISME Journal*.

[12] Hu Y., Zhou X., Liu P., Wang B., Duan D. M., Guo D. H. (2016). A comparison study of metformin only therapy and metformin combined with Chinese medicine jianyutangkang therapy in patients with type 2 diabetes: a randomized placebo-controlled double-blind study. *Complementary Therapies in Medicine*.

[13] Mu Y., Ji L., Ning G. (2016). Expert consensus on clinical application of metformin (2016 edition). *Chinese Journal of Diabetes*.

[14] Garber A. J., Duncan T. G., Goodman A. M., Mills D. J., Rohlf J. L. (1997). Efficacy of metformin in type II diabetes: results of a double-blind, placebo-controlled, dose-response trial. *The American Journal of Medicine*.

[15] Fujioka K., Brazg R. L., Raz I. (2005). Efficacy, dose–response relationship and safety of once-daily extended-release metformin (Glucophage® XR) in type 2 diabetic patients with inadequate glycaemic control despite prior treatment with diet and exercise: results from two double-blind, placebo-controlled studies. *Diabetes, Obesity and Metabolism*.

[16] Chinese Diabetes Society (2014). Chinese diabetes guidelines for type 2 diabetes. *Chinese Journal of Diabetes*.

[17] Ma J., Liu L. Y., Wu P. H., Liao Y., Tao T., Liu W. (2014). Comparison of metformin and repaglinide monotherapy in the treatment of new onset type 2 diabetes mellitus in China. *Journal of Diabetes Research*.

[18] Sin H. Y., Kim J. Y., Jung K. H. (2011). Total cholesterol, high density lipoprotein and triglyceride for cardiovascular disease in elderly patients treated with metformin. *Archives of Pharmacal Research*.

[19] Ji L., Li H., Guo X., Li Y., Hu R., Zhu Z. (2013). Impact of baseline BMI on glycemic control and weight change with metformin monotherapy in Chinese type 2 diabetes patients: phase IV open-label trial. *PLoS One*.

[20] UK Prospective Diabetes Study (UKPDS) Group (1998). Effect of intensive blood-glucose control with metformin on complications in overweight patients with type 2 diabetes (UKPDS 34). *The Lancet*.

[21] Holman R. R., Paul S. K., Bethel M. A., Matthews D. R., Neil H. A. (2008). 10-year follow-up of intensive glucose control in type 2 diabetes. *The New England Journal of Medicine*.

[22] Bolen S., Feldman L., Vassy J. (2007). Systematic review: comparative effectiveness and safety of oral medications for type 2 diabetes mellitus. *Annals of Internal Medicine*.

[23] Blonde L., Rosenstock J., Mooradian A. D., Piper B. A., Henry D. (2002). Glyburide/metformin combination product is safe and efficacious in patients with type 2 diabetes failing sulphonylurea therapy. *Diabetes Obesity and Metabolism*.

[24] Levin A., Stevens P. E., Bilous R. W. (2013). Kidney disease: improving global outcomes (KDIGO) CKD work group. KDIGO 2012 clinical practice guideline for the evaluation and management of chronic kidney disease. *Kidney International Supplements*.

[25] Lipska K. J., Bailey C. J., Inzucchi S. E. (2011). Use of metformin in the setting of mild-to-moderate renal insufficiency. *Diabetes Care*.

[26] Reinstatler L., Qi Y. P., Williamson R. S., Garn J. V., Oakley G. P. (2012). Association of biochemical B_12_ deficiency with metformin therapy and vitamin B_12_ supplements: the National Health and Nutrition Examination Survey, 1999–2006. *Diabetes Care*.

[27] Aroda V. R., Edelstein S. L., Goldberg R. B. (2016). Long-term metformin use and vitamin B12 deficiency in the diabetes prevention program outcomes study. *The Journal of Clinical Endocrinology & Metabolism*.

[28] Song J., Li J., Hou F., Wang X., Liu B. (2015). Mangiferin inhibits endoplasmic reticulum stress-associated thioredoxin-interacting protein/NLRP3 inflammasome activation with regulation of AMPK in endothelial cells. *Metabolism*.

[29] Muruganandan S., Srinivasan K., Gupta S., Gupta P. K., Lal J. (2005). Effect of mangiferin on hyperglycemia and atherogenicity in streptozotocin diabetic rats. *Journal of Ethnopharmacology*.

[30] Guo F., Huang C., Liao X. (2011). Beneficial effects of mangiferin on hyperlipidemia in high-fat-fed hamsters. *Molecular Nutrition & Food Research*.

[31] Zhang Y., Li X., Zou D. (2008). Treatment of type 2 diabetes and dyslipidemia with the natural plant alkaloid berberine. *The Journal of Clinical Endocrinology & Metabolism*.

[32] Rajasekaran S., Ravi K., Sivagnanam K., Subramanian S. (2006). Beneficial effects of *aloe vera* leaf gel extract on lipid profile status in rats with streptozotocin diabetes. *Clinical and Experimental Pharmacology and Physiology*.

[33] Zhou L., Zuo Z., Chow M. S. (2005). Danshen: an overview of its chemistry, pharmacology, pharmacokinetics, and clinical use. *The Journal of Clinical Pharmacology*.

[34] Cheng N., Ren N., Gao H., Lei X., Zheng J., Cao W. (2013). Antioxidant and hepatoprotective effects of *Schisandra chinensis* pollen extract on CCl_4_-induced acute liver damage in mice. *Food and Chemical Toxicology*.

[35] Handelsman Y., Bloomgarden Z. T., Grunberger G. (2015). American Association of Clinical Endocrinologists and American College of Endocrinology – clinical practice guidelines for developing a diabetes mellitus comprehensive care plan – 2015. *Endocrine Practice*.

[36] Hari P., Nerusu K., Veeranna V. (2012). Gender-stratified comparative analysis of various definitions of metabolic syndrome and cardiovascular risk in a multiethnic U.S. population. *Metabolic Syndrome and Related Disorders*.

